# The current status of Zika virus in Southeast Asia

**DOI:** 10.4178/epih.e2016026

**Published:** 2016-06-16

**Authors:** Viroj Wiwanitkit

**Affiliations:** Department of Public Health Curriculum, Surindra Rajabhat University, Surin, Thailand; Department of Tropical Medicine, Hainan Medical University, Haikou, Hainan, China; Faculty of Medicine, University of Niš, Niš, Serbia

**Keywords:** Zika virus, Infection, Southeast Asia

## Abstract

**OBJECTIVES:**

Zika virus currently poses a global threat and is a major public health issue throughout Latin America and the Caribbean. However, Zika virus infections in humans have also been observed in other regions, including Southeast Asia, where arboviral diseases are very common. In this study, we summarize the current status of Zika virus in Southeast Asia. This review aims to provide an overview of the current situation and also to suggest ways of adequately managing the emergence of Zika virus in Southeast Asia.

**METHODS:**

The literature searching for the reports on Zika virus in Southeast Asia was done using standard database PubMed and the re-analysis and summarization on the reports was done.

**RESULTS:**

A limited number of reports have addressed Zika virus disease in Southeast Asia, but it is has been confirmed that a problem already exists. Individual case reports and outbreaks of Zika virus have been confirmed in Southeast Asia. Several reports have also described patients becoming infected after visiting Southeast Asia. In addition, the concurrent circulation of Zika virus with other arboviruses has been confirmed.

**CONCLUSIONS:**

As a tropical region with a high prevalence of arboviral diseases, the emergence of Zika virus in Southeast Asia is a major concern. It is essential for local medical personnel to recognize this disease. Given the status of Southeast Asia as a globally important tourist destination, continuous updates on the status of Zika virus in Southeast Asia are required and should be incorporated into global health advisories regarding travel.

## INTRODUCTION

Zika virus was discovered in 1947 in Uganda, and has subsequently emerged as a new disease [1,2]. Initially, Zika virus was primarily observed in forests circulating between primates and mosquitoes [1,2]. However, its presence in humans has been sporadically reported in both Asia and Africa. From the 1960s to the 1980s, the African lineage of the virus caused sporadic infections in several African countries. Subsequently, the emerging problem due to Zika virus infection became an emerging health problem in Asia. Nevertheless, the clinical features of the disease were not serious. It seemed to cause mild infections in humans, and almost all cases involved a low fever and were self-limiting. Moreover, no outbreaks were reported. Therefore, Zika virus disease caused little concern at that time.

However, greater levels of concern regarding this infection developed due to a recent outbreak on the Yap Islands in Micronesia. The Zika virus then spread to French Polynesia and other Pacific regions, causing several outbreaks in 2013 and 2014 [3,4]. In 2015, a major outbreak in South America took place. As of 2016, it has been estimated that more than 1.5 million people in Brazil have already been infected [5]. This finding led the World Health Organization to issue a declaration emphasizing the importance of Zika virus as an emerging disease [6]. This outbreak caused considerable concern among specialists in public health when new evidence was found confirming that Zika virus infections in pregnant women can induce fetal abnormalities [7].

At present, Zika virus disease is a major public health issue throughout Latin America and the Caribbean. However, Zika virus infections have also been observed in other regions, such as Southeast Asia, where arboviral diseases are very common. However, fewer cases have been confirmed than in Latin America. Due to the clinical similarity of Zika virus infections with both dengue and chikungunya fever, which are prevalent in this area, it is possible that Zika virus infections are underdiagnosed [8]. This report summarizes the current status of Zika virus in Southeast Asia, aiming to provide an overview of the current situation and also to suggest ways of adequately managing the emergence of Zika virus in Southeast Asia.

## ZIKA VIRUS INFECTIONS IN SOUTHEAST ASIA

Southeast Asia is a tropical region where many tropical diseases are endemic. In particular, the arboviral diseases dengue and chikungunya fever are highly prevalent in this area. The main reason for this is the abundance of mosquito vectors (*Aedes* species) that are also able to transmit Zika virus; therefore, it is clear that Zika virus can be transmitted in this region and may emerge as a new major problem. Southeast Asian countries may be the next major foci of Zika virus outbreaks. The data on the present status of the disease in this area are also useful to enhance global monitoring of the disease. In this section, the situation in each Southeast Asian country is summarized.

### Indonesia

Indonesia is an island country in Southeast Asia, located close to the Pacific islands where outbreaks of Zika virus have recently been reported. The well-known Yap Islands in Micronesia are just a few thousand kilometers northwest of Indonesia. The first report of Zika virus disease in Indonesia was published in 1981 by Olson et al. [9], who presented a series of seven patients from Central Java with infected with the Zika virus. The infections were confirmed serologically, and the authors stated that “none of the seven patients had a headache or rash.” This observation is interesting, as it was recently stated that an “afebrile presentation of Zika virus disease is possible and becomes the difficulty in diagnosis” [10].

Some additional reports regarding Zika virus in Indonesia have been published. In 2012, Balm et al. [11] used a diagnostic reverse transcriptase polymerase chain reaction assay for Zika virus to study its prevalence in 88 collected serum samples and reported a prevalence of zero. However, in a recent report from Central Sumatra by Perkasa et al. [12], one of many flavivirus positive test results was finally reported to be Zika virus, confirming that Zika virus infections are a current problem in Indonesia. The authors concluded that “the isolation and characterization of Zika virus from a resident with no travel history confirm that the virus is circulating in Indonesia […] by mimicking mild dengue virus infection.” It is therefore clear that Zika virus must be included in the differential diagnosis of any patients with acute febrile illness in this area [8].

Moreover, reports have described travelers presenting with Zika infections after returning from Indonesia [13,14]. Kwong et al. [13] reported “a case of Zika virus infection in an Australian traveler who returned from Indonesia with fever and rash.” Leung et al. [14] reported another, more interesting, case of another Australian traveler who appeared to contract Zika virus by being bitten by a macaque monkey (*Macaca* species) in Indonesia. However, animal bites have never been proven to be a mode of transmission of Zika virus. The case reported by Leung et al. [14] may have also been bitten by a mosquito while visiting Indonesia.

### Singapore

Singapore is the smallest country in Southeast Asia. Similarly to other Southeast Asian countries, arboviral diseases are common in Singapore. Some interesting reports have recently described the vectors of Zika virus in Singapore [15,16]. Li et al. [15] studied Singapore’s urban *Aedes aegypti* and concluded that the mosquitoes are “susceptible to and are potentially capable of transmitting” Zika virus. Wong et al. [16] recently reported the potential of *Ae. albopictus* to transmit the virus and proposed “the possibility that the virus could be established locally.” Based on these data, strict mosquito control must be implemented in Singapore. Although no cases of human infections have been reported, Zika virus infections pose a risk in Singapore. Nevertheless, Li et al. [15] claimed that “Singapore’s current dengue virus control strategy is applicable to control Zika virus.”

### Myanmar

Myanmar is a developing country in Southeast Asia, with a high poverty rate and poor public health system. Arboviral diseases are very common in Myanmar, but few data have been published regarding their prevalence [17]. No reports have been published on Zika virus in Myanmar. The exact status of Zika virus in Myanmar remains a topic for further research.

### Lao People’s Democratic Republic

Similarly to Myanmar, the Lao People’s Democratic Republic (hereafter, Laos) is a poor country in Indochina with limited resources for public health. Arboviral diseases are also common in Laos [17]. Similarly to Myanmar, no reports have been published on Zika virus in Laos. The exact status of Zika virus in Laos remains a topic for further research.

### Cambodia

Cambodia is a country in Indochina where arboviral diseases are highly prevalent [17]. Many reports of Zika virus infections in Cambodia have been published, due to the fact that an outbreak of Zika took place in Cambodia in 2010 [18]. Many studies were carried out on isolates from this outbreak [19]. The viruses currently circulating in many Asia Pacific countries (such as Taiwan [20], Thailand [21,22], and the Cook Islands [23]) are mostly of the Asian linage and are closely related to the form of the virus found in Cambodia. Recently, Haddow et al. [24] performed a genetic epidemiology study and concluded that “the strain responsible for the Yap epidemic and the Cambodian case most likely originated in Southeast Asia.” Since Zika virus may be endemic in Cambodia, travelers to Cambodia should be aware of the possibility of coming into contact with Zika virus.

### Thailand

Thailand is a country in Indochina located next to Cambodia. Some case reports of Zika virus infections in Thailand have been published, and it was confirmed that the virus originated from Cambodia [21,22]. The disease was first identified in Thailand in 2014 [21]. Cases of Zika virus infection have been observed in several areas of Thailand. Buathong et al. [21] concluded that the Zika virus was “widespread throughout Thailand.” Wikan et al. [25] performed a seroepidemiological study and found evidence of Zika virus transmission in Thailand, as well as concurrent infections of Zika virus and other arboviral diseases, such as dengue and chikungunya fever.

Zika virus has also been reported in travelers returning from Thailand [22]. The first patient with a laboratory-confirmed Zika virus infection imported into Europe in November 2013 had traveled to Thailand [26]. Infections have also been reported in non-Western travelers returning from Thailand [27]. For example, Shinohara et al. [27] reported a Japanese patient with a Zika virus infection who had traveled to Thailand, stating that “fever causes Zika virus infection could be misdiagnosed or missed and should be considered in febrile patients with a rash, especially those returning from Thailand.” Finally, an interesting case was reported of a Cambodian returning from Thailand with a Zika virus infection [28]. Therefore, it is possible that people living in an endemic country can also become infected in another country. Additionally, a Thai patient was reported to have imported Zika virus to Taiwan, although the infection was detected at the Taipei airport [29]. This indicates that Zika virus can also be carried by travelers from an endemic country to a non-endemic country, potentially resulting in a new outbreak of the disease.

### Vietnam

Vietnam is a country in Indochina where arboviral diseases are common [17]. No reports have described cases of Zika virus infections in Vietnam, but one imported case of Zika virus infection was confirmed in South Korea in April 2016 in a patient who had spent less than four weeks in the Ho Chi Minh area.

### Brunei

Brunei is a small country in Southeast Asia. No information has been published regarding the presence of Zika virus in Brunei.

### Timor Leste

Timor Leste is a small island country in Southeast Asia. No information has been published regarding the presence of Zika virus in Timor Leste.

### Malaysia

Malaysia is a tropical country in Southeast Asia where arboviral diseases are very common. The possibility that Zika virus disease could take hold in Malaysia due to an abundance of mosquito vectors was proposed long ago [9]. The virus has been isolated from a mosquito in Malaysia [30]. The isolate in Malaysia was confirmed to be closely related to samples found in Cambodia and Micronesia [24]. A serosurvey confirmed that many Malaysians have been exposed to the virus [31]. Similarly to the situation in Thailand, an infection in a traveler returning from Malaysia to Germany has also been reported [32]. Of interest, in addition to human cases, evidence has been found of Zika virus infection in orangutans living in Malaysia [33].

### The Philippines

The Philippines are an island country in Southeast Asia, located close to the Yap Islands in Micronesia, where a major outbreak of Zika virus disease occurred. The Yap Islands are located between the Philippines and Papua New Guinea. Zika virus infections have already been confirmed in the Philippines [34]. A molecular biology study using phylogenetic analysis showed that the virus observed in the Philippines was similar to that detected in Thailand and Cambodia [22].

### The Andaman/Nicobar Islands of India

The Andaman/Nicobar Islands are geographically classified as islands in Southeast Asia. No information has been published regarding the presence of Zika virus in the Andaman/Nicobar Islands.

### Christmas Island of Australia

Christmas Island is also geographically classified as an island in Southeast Asia. No information has been published regarding the presence of Zika virus on Christmas Island.

## CONCLUSION

Although mosquito-borne illnesses are abundant in Southeast Asia, few reports have been published on Zika virus disease in this area. However, Zika virus infections have been confirmed in this region (Table 1). Zika virus has been more frequently reported in non-island countries (Thailand, Laos, Myanmar, Vietnam, Malaysia, Cambodia) than in island countries (Indonesia, Singapore, Brunei, Timor Leste, the Philippines). The situation in the regions adjacent to Southeast Asia, especially the southernmost part of China and the easternmost part of India, is also very interesting. Zika virus has not yet been reported to be present in those areas, but the likelihood of underdiagnosis is high, which poses a major public health issue [8]. The recognition of the disease by local medical personnel is a major issue. Overall, preparedness is needed. The limited amount of data from some areas implies the need for further research.

As noted, case reports and outbreaks of Zika virus infections have been confirmed in Southeast Asia. Many reports have also been published describing patients becoming infected after visiting Southeast Asia. Since many tourists visit Southeast Asia annually, awareness of this risk is important and preventive measures must be taken before travel. Given the volume of tourism to Southeast Asia, continuous updates on the status of Zika virus in Southeast Asia are necessary and should be incorporated into global health recommendations for travel.

In addition, the concurrent circulation of Zika virus and other arboviruses has been confirmed. As a tropical region with a high prevalence of arboviral diseases, the emergence of Zika virus in Southeast Asia is a major concern. Zika virus infection should be considered both in local cases and in travelers returning from Southeast Asia with clinical features of arboviral disease.

## Figures and Tables

**Table 1.** Summary of important points for each country in Southeast Asia

Country	Summary
Indonesia	The first country in Southeast Asia in which Zika virus infections have been reported
Singapore	At risk
Myanmar	Limited information
Lao People’s Democratic Republic	Limited information
Vietnam	At risk
Cambodia	A recent outbreak occurred in Cambodia. It is believed that the infection spread to nearby countries, such as Thailand
Thailand	Some local case reports have been published, as well as many reports of Zika virus infection in travelers with a history of visiting Thailand
Brunei	At risk
Timor Leste	Limited information
Malaysia	Evidence of infections has been published, as well as some reports of Zika virus infections in travelers with a history of visiting Malaysia
The Philippines	At risk
